# Human mass balance study and metabolite profiling of ^14^C-niraparib, a novel poly(ADP-Ribose) polymerase (PARP)-1 and PARP-2 inhibitor, in patients with advanced cancer

**DOI:** 10.1007/s10637-017-0451-2

**Published:** 2017-03-16

**Authors:** Lotte van Andel, Z. Zhang, S. Lu, V. Kansra, S. Agarwal, L. Hughes, M. M. Tibben, A. Gebretensae, L. Lucas, M. J. X. Hillebrand, H. Rosing, J. H. M. Schellens, J. H. Beijnen

**Affiliations:** 1 Department of Pharmacy & Pharmacology, Antoni van Leeuwenhoek Hospital / The Netherlands Cancer Institute and MC Slotervaart, P.O. Box 90440, 1006 BK Amsterdam, The Netherlands; 2 Tesaro Inc., Waltham, MA USA; 3 Division of Clinical Pharmacology, Department of Medical Oncology, The Netherlands Cancer Institute, Amsterdam, The Netherlands; 4 Division of Pharmacoepidemiology and Clinical Pharmacology, Faculty of Science, Department of Pharmaceutical Sciences, Utrecht University, Utrecht, The Netherlands

**Keywords:** Niraparib, ADME, Mass balance, Metabolites, TRA, Radiolabelled

## Abstract

Niraparib is an investigational oral, once daily, selective poly(ADP-Ribose) polymerase (PARP)-1 and PARP-2 inhibitor. In the pivotal Phase 3 NOVA/ENGOT/OV16 study, niraparib met its primary endpoint of improving progression-free survival (PFS) for adult patients with recurrent, platinum sensitive, ovarian, fallopian tube, or primary peritoneal cancer in complete or partial response to platinum-based chemotherapy. Significant improvements in PFS were seen in all patient cohorts regardless of biomarker status. This study evaluates the absorption, metabolism and excretion (AME) of ^14^C–niraparib, administered to six patients as a single oral dose of 300 mg with a radioactivity of 100 μCi. Total radioactivity (TRA) in whole blood, plasma, urine and faeces was measured using liquid scintillation counting (LSC) to obtain the mass balance of niraparib. Moreover, metabolite profiling was performed on selected plasma, urine and faeces samples using liquid chromatography – tandem mass spectrometry (LC-MS/MS) coupled to off-line LSC. Mean TRA recovered over 504 h was 47.5% in urine and 38.8% in faeces, indicating that both renal and hepatic pathways are comparably involved in excretion of niraparib and its metabolites. The elimination of ^14^C–radioactivity was slow, with t_1/2_ in plasma on average 92.5 h. Oral absorption of ^14^C–niraparib was rapid, with niraparib concentrations peaking at 2.49 h, and reaching a mean maximum concentration of 540 ng/mL. Two major metabolites were found: the known metabolite M1 (amide hydrolysed niraparib) and the glucuronide of M1. Based on this study it was shown that niraparib undergoes hydrolytic, and conjugative metabolic conversions, with the oxidative pathway being minimal.

## Introduction

Niraparib (MK-4827) is a novel, investigational poly(ADP-Ribose) polymerase (PARP) inhibitor with antitumour effect in *BRCA1* and *BRCA2* mutated cancer cells [1, 2]. Niraparib has shown promising results as a monotherapy in treatment of ovarian cancer [3] and is currently under investigation as monotherapy against other solid tumours as well as in combination with bevacizumab and pembrolizumab [4, 5]. A dose-escalation study revealed that a daily dose of 300 mg is well tolerated, has favourable pharmacological properties and antitumour activity in a broad patient population regardless of biomarker status including *BRCA1* and *BRCA2* mutations [2].

Both the US Food and Drug Administration (FDA) and the European Medicines Agency (EMA) have emphasised the importance of human absorption, metabolism and excretion (AME) studies in drug discovery and drug development, and it is now required for regulatory filing [6, 7]. The use of radiolabelled molecules is a common method used to ascertain information on the excretory routes and metabolic fate of a compound at an early stage of development [8].

Preclinical studies investigating the metabolism and disposition of niraparib in toxicological model species have shown the formation of 22 metabolites (M1 – M22) in liver microsomes and hepatocyte suspensions from humans, rats and dogs [9, 10]. This article now describes the evaluation of the mass balance of niraparib in humans, via measurement of the total radioactivity (TRA) of urine and faeces samples by liquid scintillation counting (LSC), and aims to identify the major routes of excretion as well as the metabolites formed in human plasma, urine and faeces. Moreover, this study determines the plasma concentration-time profiles of niraparib and its metabolites in humans by liquid chromatography – tandem mass spectrometry (LC-MS/MS).

## Materials and methods

### Study design

Six evaluable patients were enrolled in this open-label mass balance clinical study (ClinicalTrials.gov identifier NCT02476552) and were admitted to the study center for at least 10 days, with the 11th day being optional. On Day 1, after an overnight fast, patients received an oral dose consisting of three capsules of 100 mg radiolabelled niraparib (Quotient Clinical, Ruddington, Nottingham, UK) with a total radioactivity of 100 μCi. From the moment of administration, all urine and faeces were collected. Following completion of the study, subjects were considered eligible for continued treatment with niraparib through participation in the extension study, which involved the daily intake of 300 mg unlabelled niraparib (1 cycle =28 days). The objective of this part of the study was to evaluate the safety and tolerability of niraparib in subjects with cancer. Dose reductions were allowed twice. The study was conducted in conformance with the International Conference on Harmonisation (ICH) guidelines for Good Clinical Practice (GCP) and in accordance with the Declaration of Helsinki. The protocol was approved by the Netherlands Cancer Institute Independent Ethics Committee. All patients provided written informed consent.

### Patients

Patients aged ≥18 years with a histologically or cytologically confirmed diagnosis of metastatic or locally advanced solid tumours who had failed to respond to standard therapy, had progressed despite standard therapy, or for whom no standard therapy existed, and might benefit from treatment with a PARP inhibitor were eligible for this study. Other inclusion criteria included adequate organ function, such as bone marrow function (absolute neutrophil count ≥1500/μL, platelets ≥100,000/μL, and haemoglobin ≥9 g/dL); adequate renal function (serum creatinine ≤1.5 x upper limit of normal (ULN) or a calculated creatinine clearance ≥60 mL/min); and adequate hepatic function (total bilirubin ≤1.5 x ULN or direct bilirubin ≤1 x ULN, alanine aminotransferase (ALAT) and aspartate aminotransferase (ASAT) ≤2.5 x ULN, or ≤5 in case of liver metastases). An Eastern Cooperative Oncology Group (ECOG) performance status of 0–2 was required and adequate use of birth control was mandatory for the duration of the study. Patients were excluded from the study if they had undergone palliative radiotherapy within a week of niraparib administration; had >grade 2 toxicity from prior cancer therapy; had symptomatic uncontrolled brain or leptomeningeal metastases; had known hypersensitivity to the components of niraparib; had major surgery within 3 weeks of niraparib administration; were considered a medical risk due to a serious, uncontrolled medical disorder; or had received a platelet transfusion within 4 weeks of niraparib administration. Immunocompromised patients as well as patients with confirmed or expected hepatitis B virus (HBV), hepatitis C (HCV), or human immunodeficiency virus (HIV) were also excluded from the study. Moreover, patients were refrained from taking QTc prolonging medication, proton pump inhibitors, and H2 blockers. Pregnant women were also excluded from the study.

### Study medication

**Fig. 1:**
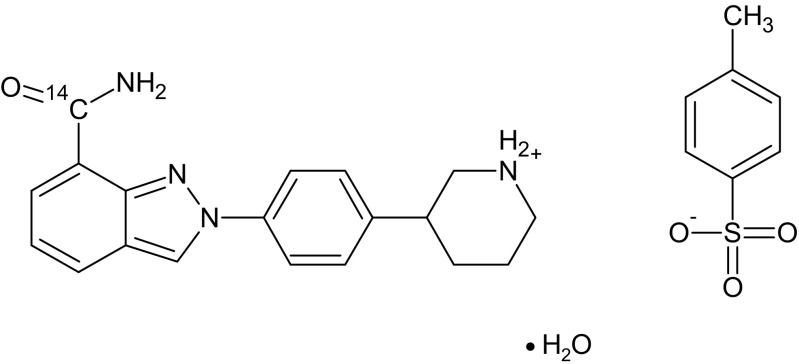
Molecular structure of ^14^C-niraparib tosylate

Radiolabelled niraparib was manufactured, packed and labelled by Quotient Clinical (Ruddington, Nottingham, UK). ^14^C–Niraparib capsules for oral administration were transparent hard gelatine capsules containing 100 mg niraparib (hot and cold) free base as the tosylate monohydrate salt (Fig. 1). Each capsule did not contain more than 1.23 MBq (33.33 μCi) ^14^C–niraparib drug substance. The oral formulation was prepared by radiodiluting a ^14^C–precursor compound to produce ^14^C–niraparib, which was subsequently combined with niraparib cold component at a ratio of 570:1 to obtain a specific activity of 0.21 μCi/mg. Appropriate amounts of the drug substance were then added to capsules, to obtain capsules containing 100 mg (33.33 μCi) niraparib. The final dose given to each patient was 300 mg (100 μCi). The chemical purity and radiochemical purity of each manufactured batch was ≥98%. Each bottle contained a single dose of 3 x ^14^C–niraparib capsules and each patient enrolled in the clinical trial was assigned one bottle. Nonlabelled niraparib was manufactured by Merck Sharpe & Dohme Corp. (Rayway, NJ, USA). Once enrolled in the extension part, patients received a bottle containing 93 capsules of unlabelled niraparib. These bottles were packed and labelled by Almac (Craigavon, UK).

### Chemicals

Acetonitrile, methanol and water (all Supra-Gradient grade) were supplied by Biosolve Ltd. (Valkenswaard, The Netherlands). Formic acid (≥98%; analytical grade), 2-propanol (>99.8%), hydrogen peroxide (30%), ethylenediaminetetraacetic acid (EDTA) 99% and sodium hydroxide (50%) were provided by Merck (Amsterdam, the Netherlands). Ammonium acetate (LC-MS grade) was purchased from Sigma Aldrich (Zwijndrecht, the Netherlands). Ultima Gold™ and Solvable™ were obtained from PerkinElmer (Groningen, the Netherlands). Water was purchased from B. Braun (Oss, the Netherlands).

### Sample collection

Blood sampling was done at the following time points: pre-dose, 1, 1.5, 2, 3, 4, 6, 9 and 12 hours (h) after administration and in the morning of Day 2, 3, 4, 5, 8, 11, 15 and 22. For each time point, a 10 mL K_2_EDTA blood collection tube was used (Vacutainer®, Becton, Dickinson and Company, Franklin Lakes, NJ, USA) and blood was drawn via a peripheral line. Whole blood aliquots were taken for radioactivity measurements, after which the tube was centrifuged (3000 rpm, 10 min, 4 °C) to produce plasma. Separate aliquots were made for radioactivity measurements as well as LC-MS/MS and metabolite profiling experiments. All samples were stored at −80 °C.

For the purpose of the mass balance study and metabolite profiling, urine and faecal samples were collected. Urine was collected at pre-determined intervals: pre-dose, 0–12 h, 12–24 h, 24–36 h and 36–48 h after administration. From Day 3 onwards, urine was collected over periods of 24 h. Urine was stored in polyethylene containers at 2–8 °C for the duration of the collection period. After each collection period, urine samples were mixed thoroughly, weighed, portioned into aliquots, and then frozen at ≤ − 70 °C. Separate aliquots were made in polypropylene tubes for analysis by LC-MS/MS, metabolite profiling and TRA analysis by LSC.

Faeces were collected and weighted per stool. Sample pre-treatment involved diluting and homogenising the faeces by adding water (1:3 *w*/*v*; B. Braun). A T25 basic Ultra Turrax (IKA Works, Staufen, Germany) was used to homogenise the samples before aliquoting. Urine and faeces sample collection occurred according to the following requirements:

1. If the total radioactivity in the Day 14 faecal/urine sample is detected to be higher than 0.1% and the total recovery of accumulative radioactivity ≤85% (faeces and urine) of the radioactivity of the dose given, faecal/urine samples will be collected every 24 h through Day 21.
2. If the total radioactivity in the Day 21 faecal/urine sample is detected to be higher than 0.1% and the total recovery of accumulative radioactivity ≤85% (faeces and urine), faecal/urine samples will continue to be collected every 24 h.
3. Faecal/urine sample collection will stop after Day 21, if the recovered radioactivity is below 1% (per 24 h) for the two consecutive days prior to Day 21 (Day 19 and Day 20). If this is not the case, collection will stop as soon as the recovered radioactivity is below 1% (per 24 h) for two consecutive days after Day 21.

### Total radioactivity analysis

TRA analysis was performed on a Liquid Scintillation Counter Tri-Carp 2910 (PerkinElmer, Groningen, The Netherlands). Aliquots of 200 μL of whole blood, plasma and faeces (all in duplicate), and 1 mL of urine (in singular) were transferred to scintillation vials. Whole blood and faecal samples needed to be moderately translucent and colourless. Therefore, 2-propanol (1 mL) was added to faecal samples to dissolve fibres, NaEDTA (0.1 mL) was added to whole blood to reduce foaming, and Solvable™ (1 mL) was added to both matrices to solubilise tissue. Hydrogen peroxide 30% (0.4 mL to faeces and 0.5 mL to whole blood) was added to reduce colour intensity. These samples were placed in a water bath (at least 40 °C; GF1086, Salm en Kipp, Breukelen, The Netherlands) to stimulate the chemical processes. Finally, 10 mL of Ultima Gold™ Cocktail (PerkinElmer) was added and the samples were analysed, either for 60 min or until the 2-sigma error was less than or equal to 5%, whichever came first. A scintillant blank was used to provide as a background sample. Results were expressed as a percentage of the administered radioactive dose and in niraparib equivalents for faeces and urine, and plasma and whole blood respectively.

### Quantification of niraparib and M1 in plasma and urine

Plasma and urine samples collected through 504 h after administration were analysed using a validated bioanalytical assay [11]. In brief, separation of niraparib and M1 was carried out using an HPLC Acquity I Class pump (Waters, Milford, MA, USA) supplied with a SunFire C18 column (50 mm × 2.1 mm, 5 μm, Waters). Gradient elution was applied using 20 mM ammonium acetate in water (mobile phase A) and 0.1% formic acid in acetonitrile-methanol (50:50, *v*/v; mobile phase B). The validated calibration range of niraparib and M1 was 1–500 ng/mL in plasma and 1–100 ng/mL in urine. Quality control samples were prepared and analysed together with the study samples. Sample analysis acceptance criteria for bioanalytical data according to the FDA and EMA guidelines [12, 13] were applied and results were reported using Analyst 5.2 software.

### Pharmacokinetic analysis

Whole blood and plasma TRA data as well as niraparib plasma concentrations were used to determine the maximum observed plasma concentration (C_max_), time to reach maximum plasma concentration following drug ingestion (t_max_), the area under the plasma concentration-time curve from time zero to time of last measurable concentration (AUC_0-last_), and if the data allowed, area under the plasma concentration-time curve from time zero to infinity (AUC_0-inf_), apparent volume of distribution during terminal phase V_d_/F, apparent total clearance of the drug from plasma after oral administration (CL/F), and the terminal phase half-life (t_1/2_)_._ Analysis was done by a non-compartmental method using WinNonlin Phoenix Version 6.2.1 or higher (Pharsight Corporation, St. Louis, Missouri, USA).

### Safety assessment

The safety of niraparib was evaluated during the study. The National Cancer Institute Common Terminology Criteria for Adverse Events (NCI CTCAE) grading system was used to describe the severity of the adverse events (AEs) that occurred. Haematology, blood chemistry and vital signs were also part of the safety assessment.

### Metabolite profiling

#### Sample selection

Metabolite profiling was divided into two parts: metabolite screening and identification, and metabolite quantification. During the first part, inter-patient pooled samples were analysed to screen for the presence of metabolites. Afterwards, individual patient samples or within-patient pooled samples (depending on the matrix) were analysed and metabolites found during the screening phase were quantified. Plasma, urine and faeces samples were selected both for screening and quantification. Three time points were selected for screening of plasma samples. Besides the pre-dose, a sample around the t_max_ was chosen, because these samples contained the highest amount of radioactivity. It was also of interest to select a sample in which the amount of radioactivity was much higher than the amount of parent compound, because this is indicative of the presence of metabolites. Consequently, 4 h and 48 h were selected and screened for metabolites. Individual patient samples from 0 h, 4 h, 12 h, 24 h, 48 h, 72 h and 168 h were analysed and metabolites in these samples were quantified.

All urine samples were pooled within patients, and afterwards these samples were pooled between patients. For metabolite screening, the pre-dose, 0–24 h and 24–72 h were pooled between patients and analysed. 24 h–intervals were also pooled together within patients and a pre-dose, 0–24 h, 24–72 h and 72–144 h were analysed.

The same procedure was applied for faeces samples. Again, 24 h-interval pools were made, first within patients and then between patients. The latter were used for metabolite screening, whereas the first were analysed afterwards, during the quantification step. The time points that were chosen included a pre-dose, 0–24 h, 24–72 h and 72–144 h, for both screening and quantification.

#### Preparation of biological samples

##### Plasma

Plasma samples (pre-dose, 4 h and 48 h) were pooled by equal volumes. In addition, the individual samples (pre-dose, 4 h, 12 h, 24 h, 48 h, 72 h and 168 h) were analysed. Plasma samples were extracted once with acetonitrile-methanol (50:50, *v*/v). The obtained supernatant was evaporated to dryness and the dried supernatant was reconstituted in 10 μL 0.1% formic acid in acetonitrile and 90 μL 20 mM ammonium acetate in water. An aliquot of the clear supernatant was analysed for total radioactivity to determine extraction efficiency. The remaining supernatant was used for analysis by LC-MS^n^ in combination with off-line LSC (LC-LSC-MS^n^).

##### Urine

Urine samples were first pooled within patients. Three pools (0–24 h, 24–72 h and 72–144 h) were prepared, after which pools across patients were made. These samples were analysed without pre-treatment.

##### Faeces

Homogenised faeces samples were pooled, by equal percentage of weight, across all subjects for selected time periods (pre-dose, 0–24 h, 24–72 h and 72–144 h). Faeces homogenate samples were extracted once with 0.1% formic acid in acetonitrile. The obtained supernatant was evaporated to dryness and the dried supernatant was reconstituted in 75 μL acetonitrile and 75 μL 20 mM ammonium acetate in water. An aliquot of the clear supernatant was analysed for total radioactivity to determine extraction recovery. The remaining supernatant was used for LC-LSC-MS^n^ analysis.

#### LC-MS/MS systems

Samples were analysed by LC (Shimadzu, Kyoto, Japan) coupled to a linear ion trap mass spectrometer (Thermo Electron, Waltham, MA, USA) in combination with off-line LSC (LC-Linear Ion Trap LTQ XL Mass Spectrometry (MS)-LSC). To separate known and unknown metabolites, an HPLC gradient was applied. A flow rate of 1 mL/min was maintained during the following 50-min gradient: starting at 10%B and increasing to 36.25%B over 35 min, then to 90%B in 12 min. This was held for 2 min and then it was reverted back to the initial conditions of 10%B at 49 min. Mobile phase A consisted of 20 mM ammonium acetate in water and mobile phase B consisted of 0.1% formic acid in acetonitrile. Structural information on niraparib metabolites was obtained using Data Dependent Acquisition. Accurate mass measurements were performed to confirm the chemical structures of the metabolites. These measurements were performed on an Ultimate 3000 LC (Dionex) coupled to an LTQ Orbitrap XL MS (Thermo Electron, Waltham). The same gradient composition was applied. Additional LC-MS/MS settings can be found in Tables 1 and 2.

**Table 1:** HPLC settings

Pump	LC-20 AD (Shimadzu)		Ultimate 3000 RSLC nanoSystem (Dionex)	
Flow rate	1.0 mL/min		12 μL/min	
Analytical column	Phenomenex Synergi Hydro-RP (250 × 4.6 mm, 4 μm)		Phenomenex Synergi Hydro-RP 80A (150 × 0.5 mm, 4 μ)	
Column oven	35 °C		Ultimate 3000 RSLC NanoSystem	
Run time	50 min		60 min	
Mobile phase A	20 mM ammonium acetate in H_2_O		20 mM ammonium acetate in H_2_O	
Mobile phase B	0.1% HCOOH in ACN		0.1% HCOOH in ACN	
Gradient composition	Time (min)	Mobile phase B (%)	Time (min)	Mobile phase B (%)
	0.0	10	0.0	10
	0.1	10	0.1	10
	35.0	36.25	35.0	36.25
	47.0	90	47.0	90
	49.0	90	49.0	90
	49.1	10	50.0	10
	50.0	10	52.0	10
			55.5	90
			57.0	10
			60.0	10
Autosampler	SIL-HTc (Shimadzu)		Ultimate 3000 RS autosampler (Dionex)	
Tray temperature	4 °C		4 °C	
Injection volume	0.050 mL (U + P); 0.010 mL (F)		1 μL	
Splitter	Accurate (LC packings)		NA	
Split ratio (collector:MS)	1:3		NA

**Table 2:** MS settings

Mass spectrometer	LTQ XL (Thermo Electron)	LTQ Orbitrap discovery (Thermo scientific)
Ionization/interface	ESI, positive ionization mode	ESI
Scan range	50–1100	80–1100
Valve	0–2 min (waste); 2–35 min (source); 35–50 min (waste)	Not used
Sheath gas flow	30 arb	15 arb
Aux gas flow	15 arb	0 arb
Sweep gas flow	5 arb	0 arb
Spray voltage	5 kV	5 kV
Normalised collision energy	35 V	32 V
Capillary Temperature	375 °C	270 °C
Capillary Voltage	40 V	46 V
Tube Lens	75 V	125

#### Metabolite screening and identification

The metabolite profiles of ^14^C–niraparib-derived radioactivity were determined for selected aliquots of extracts of plasma, urine and faecal homogenates using LC-MS/MS, fractionation and off-line LSC. After pre-treatment, samples were injected onto a Phenomenex Synergi Hydro-RP (250 × 4.6 mm, 4 μm) column. Data Dependent Acquisition was applied, causing the detection and fragmentation of the most abundant ions. Besides this, a parent list with possible metabolites was constructed, meaning that apart from the most abundant ions, also the ones with the masses in this parent list were further fragmentised for which MS^2^ and MS^3^ spectra were obtained. In between the HPLC and the MS a fraction collector was placed, which collected 3/4th of the flow. The remaining sample was analysed by MS. This fraction collector was set at 1 fraction per minute, in other words 50 fractions were collected per sample. LSC (Tri-Carb 4910 TR; PerkinElmer, Waltham) was performed by adding Ultima Gold™ scintillation cocktail to a sample followed by scintillation counting for 20 min. The limit of detection (LOD) and lower limit of quantification (LLOQ) were calculated based on the formulas described in “Calculations” section. The result from analysis by LSC was a radiochromatogram, showing radioactive fractions, which could be correlated directly with the retention time of the peaks in the LC chromatogram. Metabolites were identified based on accurate masses of protonated molecular ions and their fragmentation patterns. Authentic standards, when available, were used to compare chromatographic retention times and fragmentation patterns. Metabolite names were derived from Zhang et al. [9, 10], except in the case of newly discovered metabolites. The numbers designated in the radiochromatograms were given in accordance with the retention time. Each metabolite has therefore two names: a chronological number and an M-name (or a descriptive name in the case of newly identified metabolites).

#### Metabolite quantification

Individual samples (in case of plasma) and individually pooled samples (urine and faeces) were analysed in the same manner as mentioned before. Here, metabolites identified during screening were quantified and expressed as a percentage of the administered dose, in the case of urine and faeces, and as a percentage of the AUC_0-168h_ from the total amount of ^14^C administered in the case of plasma.

#### Calculations

For radioactivity analysis by LSC, the LOD and LLOQ were determined using the equations below.

1 $$ \mathrm{LOD}=\frac{2.71}{\mathrm{TE}}+4.65\sqrt{\frac{\mathrm{B}}{\mathrm{TE}}} $$

LOD: Limit of detection (DPM)
T: Counting time (min)
E: Counting efficiency
B: Background radioactivity (DPM)

With a counting time of 20 min, an observed background of 11.0 disintegrations per minute (DPM) and a counting efficiency of 93%, the LOD is 3.7 DPM, which is rounded to 4 DPM.

2 $$ \%2\mathrm{S}=\frac{200\sqrt{\left({\mathrm{CPM}}_{\mathrm{a}}+{\mathrm{CPM}}_{\mathrm{b}}\right)/\mathrm{T}}}{{\mathrm{CPM}}_{\mathrm{a}}-{\mathrm{CPM}}_{\mathrm{b}}} $$

%2 s: 2 times the standard deviation of the count expressed as percentage of counts
CPM_a_: Total CPM of a sample
CPM_b_: CPM of the background
T: Counting time (min)

At the LLOQ the %2 s value should not exceed 20%. Thus, with an observed background CPM_b_ of 10.2, a %2 s value of 20 and a counting time of 20 min, the CPM_a_ at the LLOQ is 23.2 CPM. With a counting efficiency of 93%, this corresponds to a total LLOQ of 24.9 DPM. After subtraction of the background the corrected activity at the LLOQ is 14 DPM.

## Results

### Patients

Six female patients, three with ovarian cancer, two with breast cancer and one with colorectal cancer, were enrolled in the mass balance study, with a median age of 55.5 (range 39–69), a mean weight of 73.6 kg (range 64.3–92.3) and a mean height of 167.8 cm (range 159–176). Baseline characteristics can be found in Table 3. All patients completed the first part of the study. Five were eligible for the extension study and received daily treatment with niraparib. All subjects enrolled in the extension study completed at least 2 cycles. Two patients have completed 7 or more cycles at the cut-off date. All five patients received a dose reduction at some point due to the occurrence of an adverse event.

**Table 3:** Baseline characteristics

Characteristic	Value
Age (Years)	
Mean (StdDev)	55.5 (11.10)
Median	56.5
MIN, MAX	39, 69
Sex, N (%)	
Female	6 (100)
Race, N (%)	
White	6 (100)
Ethnicity, N (%)	
Not Hispanic Or Latino	6 (100)
Smoking Status, N (%)	
Current	1 (17)
Former	3 (50)
Non-smoker	2 (33)
Illicit Drug Abuse, N (%)	
No	6 (100)
Chronic Alcohol Use, N (%)	
No	6 (100)

### Safety

At least one treatment-emergent adverse event (TEAE) was reported for each patient enrolled in the study, with a total of 21 AEs reported. Gastrointestinal disorders occurred in four patients and consisted of constipation, dyspepsia, dry mouth, nausea, abdominal distension and ascites. Furthermore, dermatitis acneiform occurred in two patients and fatigue, erysipelas, ASAT increase, myalgia, insomnia and pleuritic pain were experienced by one patient. Six of these AEs were considered likely to be related to study treatment, including constipation, dry mouth, nausea, dermatitis acneiform and ASAT increase. All of these were grade 2 or lower and no TEAEs led to study drug discontinuation or death.

### Pharmacokinetics

**Fig. 2:**
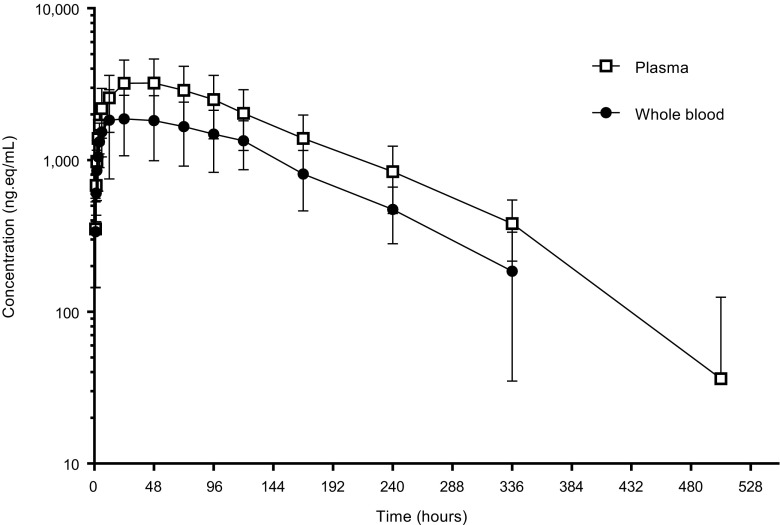
Mean (±SD) log-linear concentration-time profile of total radioactivity (niraparib-related compounds) in whole blood and plasma after a single dose of 300 mg ^14^C–niraparib to patients with advanced cancer (*n* = 6)

A summary of pharmacokinetic parameters of ^14^C–radioactivity in plasma and whole blood, unlabelled niraparib, and unlabelled major metabolite M1 in plasma is presented in Table 4. Mean total ^14^C–radioactivity (expressed as nanograms-equivalent per millilitre; ng.eq/mL) in whole blood and plasma, and niraparib and M1 in plasma concentration-versus-time data are shown in Fig. 2. Niraparib plasma concentrations peaked at 2.49 h post-dose, with a mean maximum concentration of 540 ng/mL. Mean t_½_ for unlabelled niraparib in plasma was 87.4 h, meaning that elimination of niraparib took place slowly. Niraparib CL/F was approximately 17.2 L/h and the apparent V_d_/F was 2170 L, indicating niraparib distributes extensively into tissue.

**Table 4:** Summary of pharmacokinetic parameters of ^14^C radioactivity in plasma and whole blood, unlabelled-niraparib and unlabelled major metabolite M1

Parameter		Total ^14^C- radioactivity plasma (*n* = 6)	Total ^14^C- radioactivity whole blood (*n* = 6)	Unlabelled niraparib plasma (*n* = 6)	Unlabelled M1 plasma (*n* = 6)
C_max_^a^ (ng/mL)	Mean	3260	2110	540	476
	CV%	42.4	48.3	30.5	39.4
t_max_ (h)	Median	48.02	24.02	2.49	9.02
	Range	23.98–48.40	3.00–72.03	1.52–5.98	5.98–24.20
AUC_0-last_^b^ (μg*h/mL)	Mean	551	313	18.4	40.8
	CV%	44.8	44.7	29.9	42.1
AUC_0-inf_^b^ (μg*h/mL)	Mean	594	348	18.5	41.2
	CV%	43.0	41.8	29.6	42.3
t_1/2_ (h)	Mean	92.5	90.5	87.4	78.4
	CV%	8.6	9.0	19.1	17.2
CL/F (L/h)	Mean	0.601	1.01	17.2	NA
	CV%	47.9	44.3	26.1	NA
V_d_/F (L)	Mean	79.7	130	2170	NA
	CV%	49.1	41.2	32.2	NA

*NA* Not Applicable

*AUC*_*0-inf*_ Area under the plasma concentration-time curve from time 0 to infinity, *AUC*_*0-last*_ Area under the plasma concentration-time curve from time 0 to the last quantifiable concentration, *CL/F* Apparent oral clearance, *C*_*max*_ maximum observed plasma concentration, *CV* Coefficient of variation, *NA* Not applicable; *t*_*max*_ Time to reach maximum observed plasma concentration, *t*_½_ Terminal half-life, *V*_*d*_ */F* Apparent oral volume of distribution

^a^C_max_ unit for ^14^C–radioactivity is ng equivalent/mL

^b^Unit for AUCs for total ^14^C–radioactivity is µg equivalent*hr./mL

Pharmacokinetic analysis for ^14^C–radioactivity in whole blood and plasma showed that the amount of radioactivity in plasma is 1.7 higher than in whole blood. This suggests that niraparib metabolites are distributed more in plasma than in red blood cells.

### Mass balance

**Fig. 3:**
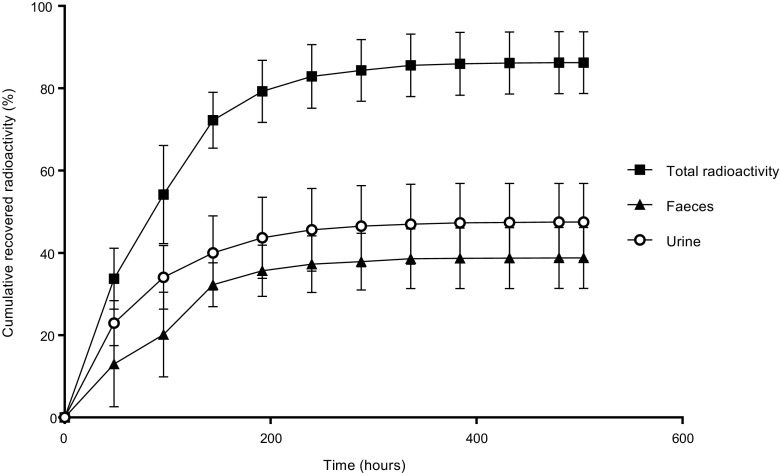
Mean (±SD) cumulative recovered radioactivity in excreta after a single dose of 300 mg ^14^C–niraparib to patients with advanced cancer (*n* = 6)

As shown in Fig. 3, over 50% of the administered dose was already recovered within the first 4 days (96 h) after dosing. A mean total of 86.3% of the administered radioactive dose was recovered in excreta through 504 h post-dose, of which 47.5% was recovered in urine and 38.8% was found in faeces. These data demonstrate that both the hepatic/biliary route and renal clearance are the predominant excretion routes for niraparib and its metabolites. Collection of faeces and urine occurred in accordance with the criteria mentioned in “Sample collection” section. This meant that collection was stopped after 21, 12, 10, 22, 17 and 11 days, for patients 1, 2, 3, 4, 5 and 6, respectively.

### Metabolites in plasma

**Fig. 4:**
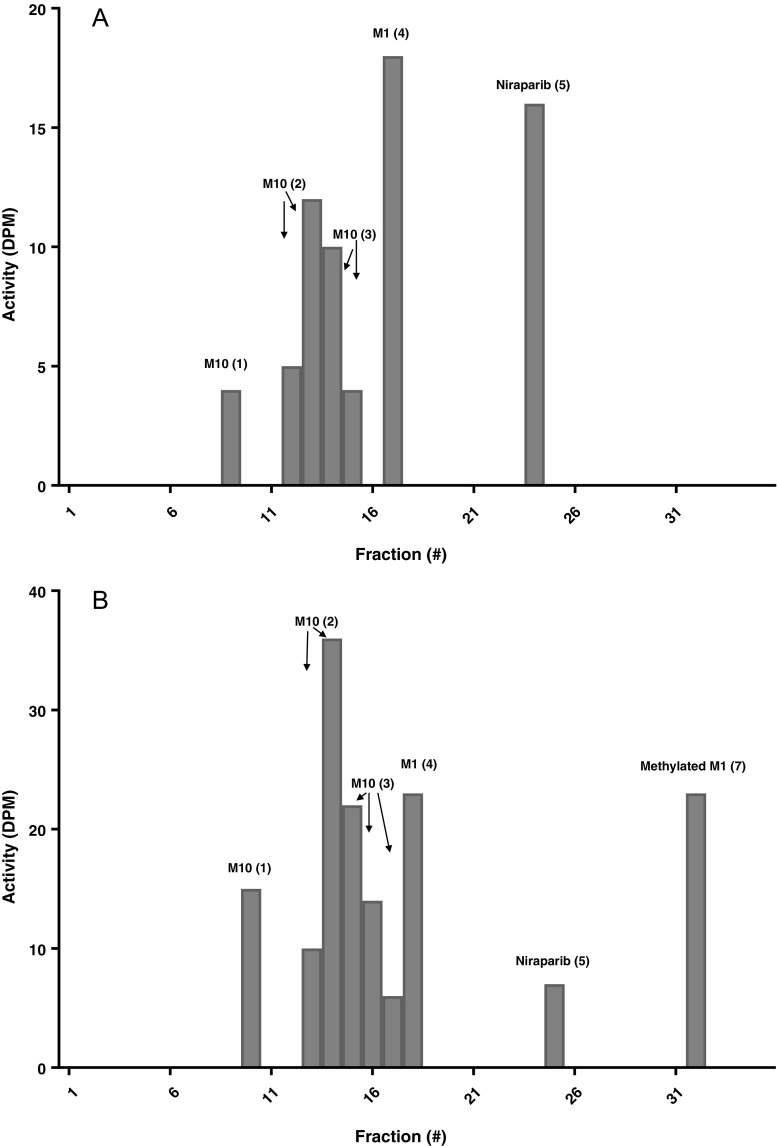
Radiochromatogram of plasma screening samples collected 4 h (**a**) and 48 h (**b**) post-dose. Note that the scale of the y-axis is different in each radiochromatogram.

Figure 4 shows the radiochromatograms for the plasma screening samples. Peaks are numbered in chronological order i.e. in order of retention time. Six compounds were detected in the plasma matrix and quantified. Concentrations were expressed in niraparib ng.eq/mL and ultimately AUC_0-168h_ values were calculated. These values were compared to the total ^14^C–AUC_0-168h_ and finally expressed as a percentage of the ^14^C–AUC_0-168h_. The total radioactivity measured in the fractions of these samples was low (up to 35 DPM). As expected, unchanged parent drug concentrations were lower in the 48-h sample and higher concentrations of metabolites were found as compared to the 4 h–sample. Niraparib (5) accounted for only 2.4% of the relative ^14^C–AUC_0-168h_. M1 (4) accounted for 9.3%, and unknown metabolites for the majority (58.2%) of the radioactive dose. Of this, 55.7% was attributed to the M1 glucuronide (1), (2) and (3), and 2.5% was accounted for by Methylated M1 (7). Finally, 1.1% of the administered dose could not be accounted for in the radiochromatograms. A total of 69.9% of the total radioactivity was explained and the remaining radioactivity was not recovered due to sample pre-treatment loss, which was 29%.

### Metabolites in urine

**Fig. 5:**
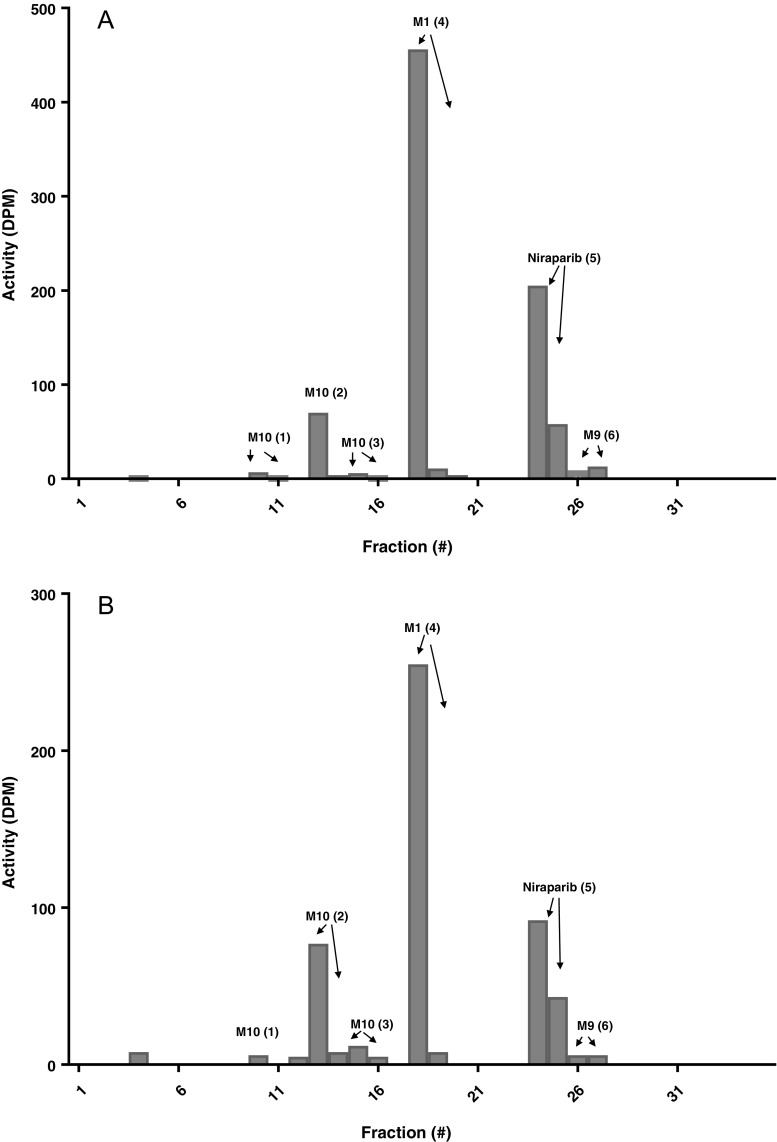
Radiochromatogram of urine screening samples collected 0-24 h (**a**) and 24-72 h (**b**) post-dose. Note that the scale of the y-axis is different in each radiochromatogram.

Figure 5 shows the radiochromatograms from pooled urine samples. The profile did not change considerably over time. The predominant peaks found in urine were niraparib (5), M1 (4) and M10 (2), which accounted for 10.5, 20 and 6.2% of the administered dose, respectively. A total of 36.7% of the administered dose was explained by these compounds. A very small amount of the other glucuronidated forms of M1 (1) and (3) as well as M9, monooxygenated dehydrogenated niraparib (6), were detected, but were considered low abundant metabolites because of their low concentrations. A small portion of 3.3% of the administered dose was left unaccounted for and could not be identified.

### Metabolites in faeces

**Fig. 6:**
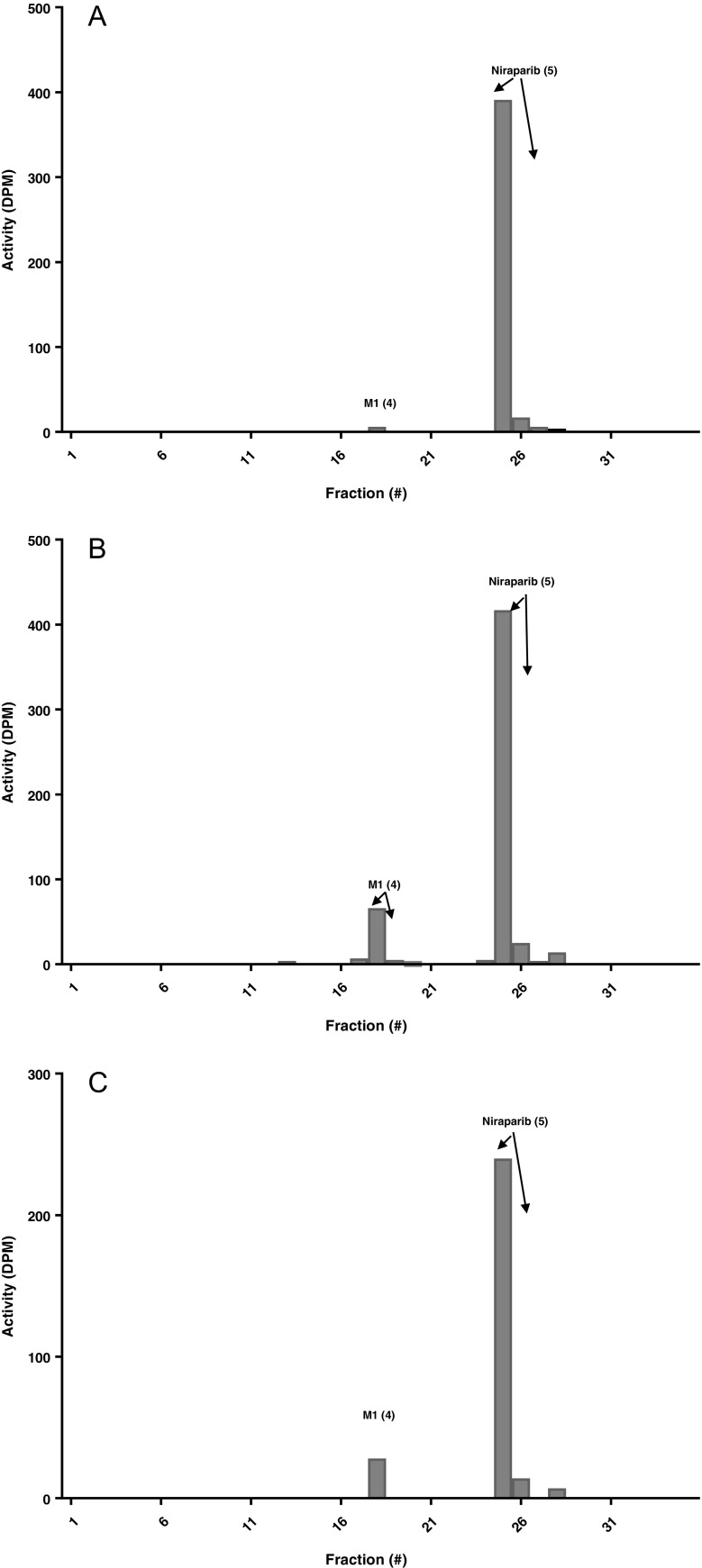
Radiochromatogram of faeces screening samples collected 0-24 h (**a**), 24-72 h (**b**) and 72-144 h (**c**) post-dose. Note that the scale of the y-axis is different in each radiochromatogram.

Figure 6 shows the radiochromatograms from pooled faeces samples. The profile was very similar in both selected samples, with only two compounds detected. The majority was assigned to niraparib (5), accounting for 18.7% of the administered dose, whereas only 2.4% was excreted via faeces as M1 (4). A small fraction of 1.2% of the radioactivity could not be accounted for in the radiochromatograms and 9.3% was lost during sample pre-treatment.

### Metabolite identification

The structural characterisation and identification of all metabolites is described below. An overview of all metabolite characteristics can be found in Table 5.

**Table 5:** Summary of niraparib metabolite profiling

Compound			Plasma exposure^a^	Amount excreted^a^							
Compound (ID)	RT (min)	(Proposed) identity	Relative AUC_0-168h_ (% of AUC_0-168h_ from total ^14^C)	Urine (0–144 h)	Faeces (0–144 h)	Total (0–144 h)	Experimental [M + H]^+^ *m/z*	Theoretical [M + H]^+^ *m/z*	Mass accuracy *ppm*	Formula	Fragment ions
M10 (1)	9	M1-glucuronide	6.0%	<LLOQ	ND	ND	498.18597	498.187642	-3.36	C_25_H_29_N_3_O_8_	304, 276, 263, 235, 207
M10 (2)	12	M1-glucuronide	26.8%	6.2%	ND	6.2%	498.18588	498.187642	-3.54	C_25_H_29_N_3_O_8_	322, 304
M10 (3)	14	M1-glucuronide	22.9%	<LLOQ	ND	ND	498.18564	498.187642	-4.02	C_25_H_29_N_3_O_8_	304, 276, 263, 235, 207
M1 (4)	17	Amide hydrolysed niraparib	9.3%	20.0%	2.4%	22.4%	322.15479	322.155552	-2.37	C_19_H_19_N_3_O_2_	304, 276, 263, 235, 207
Niraparib (5)	25	Parent drug	2.4%	10.5%	18.7%	29.2%	321.17062	321.171536	-2.85	C_19_H_20_N_4_O	304, 276, 263, 235, 207
M9 (6)	26	Mono-oxygenated dehydrogenated M1	ND	<LLOQ	ND	ND	336.13385	336.134817	-2.88	C_19_H_17_N_3_O_3_	318, 290, 273, 261, 245, 231
Methylated M1 (7)	31	Methylated M1	2.5%	ND	ND	ND	336.17032	336.171202	-2.62	C_20_H_21_N_3_O_2_	304, 276, 263, 235, 207
Total assigned radioactivity after fractionation			69.9%	36.7%	21.1%	57.8%					
Total radioactivity in matrix			NA	40.0%	31.6%	71.6%					
Loss during pre-treatment			29.0%	NA	9.3%	9.3%					
Unaccounted for in radiochromatogram			1.1%	3.3%	1.2%	4.5%					

^a^Fractions < LOD (4 DPM) were regarded as containing 0 DPM

*ND* Not Detected (<LOD (4 DPM)), *NA* Not Applicable, *RT *retention time

#### Niraparib (5)

The identity of niraparib was confirmed by comparing the obtained mass spectra and retention time with the mass spectra and retention time of the reference standard. The MS^2^ spectra showed a product ion of *m/z* 304, which corresponds to the loss of NH_3_. The MS^3^ spectra also showed *m/z* 276, which indicates an additional loss of 28, and can be attributed to the loss of CO.

#### M1 (4)

M1 was identified similarly to niraparib. Reference standards were available and MS/MS^2^/MS^3^ spectra from these reference standards and observed peaks in the chromatogram showed strong similarities. Moreover, the retention times were identical. The MS^2^ spectra showed a product ion of *m/z* 304, which corresponds to the loss of H_2_O. The MS^3^ spectra is identical to the niraparib spectra. It is therefore confirmed that niraparib and M1 have identical fragmentation patterns.

#### M10 (1), (2) and (3)

The MS spectra of metabolites (1), (2) and (3) are similar and show a parent protonated mass of *m/z* 498. In the MS^2^ spectra two ions were detected: *m/z* 322, the mass of M1 (4), (after the loss of 176 Da, indicating the presence of a glucuronide group) and *m/z* 304 (an additional loss of H_2_O). The MS^3^ spectra of metabolites (1), (2) and (3) show similar ions as detected for M1 (4). These data support the identification of M1 glucuronides (M10). Compounds (1) and (3) showed additional product ion *m/z* 480 in the MS^2^ spectra. The loss of 18 Da could indicate a loss of H_2_O. This could suggest *N*-glucuronides rather than *O*-glucuronides. However, the position of the glucuronide group could not be confirmed solely based on these data.

#### Methylated M1 (7)

Compound (7) shows a parent ion of *m/z* 336. MS^2^ and MS^3^ spectra show similar patterns as niraparib (5), M1 (4) and M10 (1) and (3), with product ion *m/z* 304 and a fragmentation pattern with *m/z* 263, 235 and 276. It was, based on the high resolution mass data, confirmed as a methylated M1. Even though methylations belong to the less common phase II biotransformations, these types of reactions have found to occur especially for substrates that contain -C, −N, −O and -S functional groups [14]. This results, in contrast to most other phase II conjugation reactions, in more hydrophobic metabolites. This conclusion is supported by the fact that this compound elutes later than all other metabolites, which can be explained by the reduction of hydrophilicity by binding to the O-functional group, thus making the compound less polar [15].

#### M9 (6)

Compound (6) shows a protonated ion at *m/z* 336. The product ion of *m/z* 318 likely results from a loss of H_2_O. The additional loss of CO produces a fragment of *m/z* 290. This again can be fragmentised to eventually a product with *m/z* 231, due to subsequent losses of NH_3_, CH_3_N, CH_3_NO and C_2_H_5_NO.

## Discussion

**Fig. 7:**
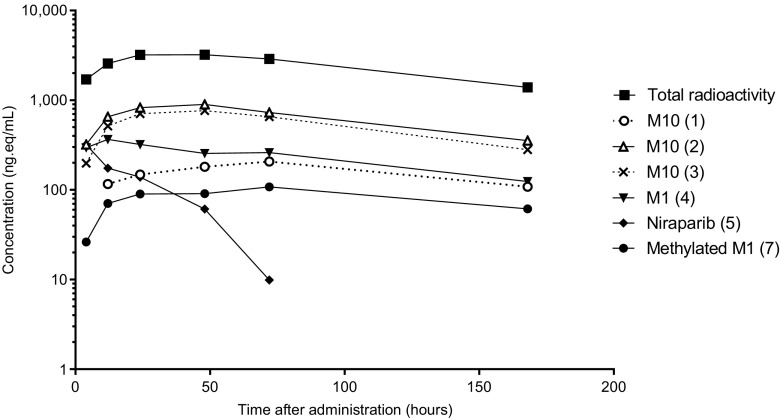
Mean log-linear concentration-time profiles of radioactive metabolites found in plasma between 0 and 168 h

This was a mass balance study conducted in six female volunteers with various types of cancer at a single study center, and it investigated the absorption, metabolism and excretion of ^14^C–niraparib after a single oral dose of 300 mg. The niraparib C_max_ concentration of 540 ng/mL was observed at 2.49 h post-dose (t_max_). Niraparib was eliminated biphasically with a mean t_1/2_ of 87.4 h. The t_1/2_ for total radioactivity in plasma was slightly longer, which is explained by the longer t_1/2_ of niraparib metabolites. A plasma-concentration time curve of niraparib and metabolites can be found in Fig. 7. The apparent clearance was approximately 17.2 L/h. The plasma:whole blood ratio of 1.7, based on AUC_0-inf_ of the total radioactivity illustrates that metabolites distribute more in plasma than in red blood cells. This is consistent with red blood cell partitioning experiments done previously (data unpublished). It is important to note that it is the metabolites that distribute more into red blood cells, while niraparib penetrates more into tissues. This finding could be an important differentiation factor for niraparib efficacy.

The apparent V_d_/F of niraparib in humans, while consistently high, has shown variations across the clinical studies. For example, in the patients in Part 1of this AME study designated for the determination of absolute oral bioavailability, the apparent V_d_/F was determined to be 1220 L (data not shown). Moreover, based on the population pharmacokinetic analysis on all the patients in NOVA study [3], the apparent V_d_/F was 1074 L. Collectively, it would be conceivable to conclude that the apparent V_d_/F for niraparib appears to be quite high, at least 1000 L in cancer patients.

**Fig. 8:**
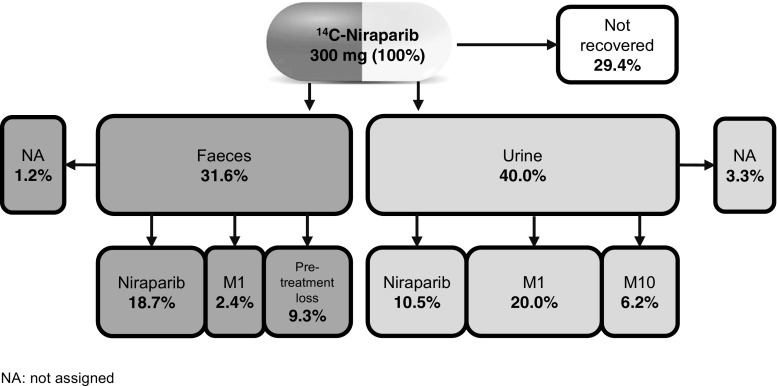
Summary of niraparib metabolite excretion through 144 h after a single oral dose of ^14^C-niraparib

The main elimination routes of niraparib and its metabolites are both the hepatic/biliary and the renal routes. Mean total radioactivity recovered in urine and faeces was 86.3% (71.1 - 91.0%) of the total administered dose, of which 47.5% (33.4 - 60.2%) was recovered in urine and 38.8% (28.3% - 47.0%) in faeces through 504 h post-dose. Metabolite profiling was done for plasma samples up to 168 h and for urine and faeces samples up to 144 h. Up to 144 h, a total of 71.6% of the administered dose was recovered in excreta, 40 and 31.6% in urine and faeces, respectively. A summary can be found in Fig. 8.

**Fig. 9:**
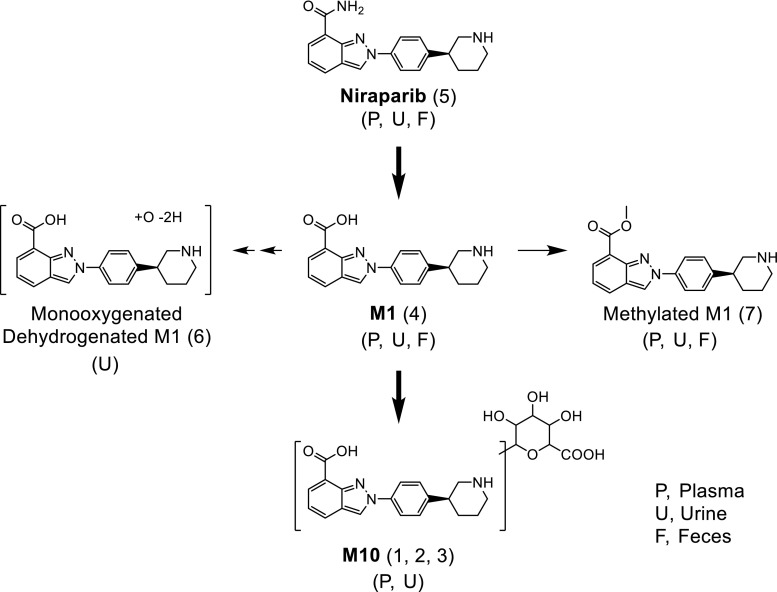
Proposed metabolic pathway of niraparib in humans

The metabolite profiles obtained by LC-LSC-MS^n^ analysis revealed that unchanged niraparib (5) was the predominant radioactive component in faeces, whereas M1 (4) was the main radioactive metabolite in urine. The glucuronidated form of M1, M10 (1), (2) and (3) was the main circulating metabolite in plasma and was considered the only major metabolite in addition to M1. Minor metabolites included M9 (6) and Methylated M1 (7). The chemical structures of these metabolites were confirmed by high resolution techniques, with mass spectra accuracy within 5 ppm. From these data it can be concluded that niraparib undergoes hydrolysis and conjugation, with the oxidative pathway seen in vitro being minimal [9]. A proposed metabolic pathway can be found in Fig. 9.

M10 (1), (2) and (3) was largely present in plasma and was only eliminated via the urine. The minor metabolites M9 (6) and methylated M1 (7) were also detected, of which the latter was quantified in plasma and not detected in urine. M9 was not detected in plasma, but was quantified in urine, albeit it being a low abundant metabolite. Metabolite M10 was a major metabolite and was found in three forms (1), (2) and (3). Based on MS data, it cannot be concluded what the position of the glucuronide on M1 is, although it could be suggested that in the case of (1) and (3), *N*-glucuronides are more likely, due to the detection of *m/z* 480 in the MS^2^ spectrum.

Upon comparing the metabolism data reported here with the data obtained from in vivo and in vitro studies [9, 10] it becomes apparent that not all metabolites detected in the animal studies have been detected in human plasma and/or excreta. For instance, the glucuronide of niraparib (M20), was not detected during the metabolite profiling of this study, nor were M2 (mono-oxygenated niraparib) and M8 (carbonyl of niraparib), whereas they were identified in human hepatocytes.

It has been demonstrated that both carboxylesterases (CEs) and CYP enzymes play a role in Phase 1 metabolism of niraparib in vitro. However, CE-mediated amide hydrolysis of niraparib to form M1 has been evidently shown as virtually the only primary pathway in cancer patients in this study, suggesting the minimal role of CYPs in niraparib metabolism.

An adequate amount of faecal and plasma samples was lost during sample clean-up, which was demonstrated by loss of radioactivity in each analysed sample. Therefore, it is impossible to rule out the potential losses of (minor) metabolites. When a trend is seen over time, the conclusion could be that the losses are compound-specific. For instance, if the amount of radioactivity lost increases with time (i.e. with increasing concentration of an analyte), and then decreases again (with decreasing concentration of that analyte), this could be indicative of losses of this one specific analyte. Such a trend was not observed and therefore it cannot be concluded that these losses were compound-specific. Nevertheless, no conclusions can be drawn about whether the compounds in the sample were lost to the same extent. Efforts were made to improve extraction recovery, but were hampered by the limited amount of plasma samples available. After achieving 70% extraction recovery, it was decided to stop the developmental phase in order to ensure that sufficient amounts were left for the experimental phase. Faeces sample pre-treatment improved by changing extraction solvents and by dissolving dried extracts into the organic phase before adding the aqueous phase. This increased extraction efficiency from approximately 20 to 90%.

In conclusion, it was shown that niraparib is moderately metabolised in humans via hydrolytic and conjugative pathways. 31.6% of the total administered dose was recovered in faeces and 40.0% was excreted in urine 144 h post-dose. Unchanged niraparib accounted for 29.9% of the dose excreted in urine and faeces. Furthermore, M1 (2.4%) was detected in faecal samples, and M1 (20.0%) and M10 (6.2%) were quantified in urine. The high volume of distribution and long elimination half-life seen in this study may be consistent with the anti-cancer activity of niraparib.
